# Neural Network Model of Memory Retrieval

**DOI:** 10.3389/fncom.2015.00149

**Published:** 2015-12-17

**Authors:** Stefano Recanatesi, Mikhail Katkov, Sandro Romani, Misha Tsodyks

**Affiliations:** ^1^Department of Neurobiology, Weizmann Institute of ScienceRehovot, Israel; ^2^Janelia Farm Research Campus, Howard Hughes Medical InstituteAshburn, VA, USA; ^3^Department of Neurotechnologies, Lobachevsky State University of Nizhny NovgorodNizhny Novgorod, Russia

**Keywords:** attractor neural networks, recall, oscillations, memory, neural representations

## Abstract

Human memory can store large amount of information. Nevertheless, recalling is often a challenging task. In a classical free recall paradigm, where participants are asked to repeat a briefly presented list of words, people make mistakes for lists as short as 5 words. We present a model for memory retrieval based on a Hopfield neural network where transition between items are determined by similarities in their long-term memory representations. Meanfield analysis of the model reveals stable states of the network corresponding (1) to single memory representations and (2) intersection between memory representations. We show that oscillating feedback inhibition in the presence of noise induces transitions between these states triggering the retrieval of different memories. The network dynamics qualitatively predicts the distribution of time intervals required to recall new memory items observed in experiments. It shows that items having larger number of neurons in their representation are statistically easier to recall and reveals possible bottlenecks in our ability of retrieving memories. Overall, we propose a neural network model of information retrieval broadly compatible with experimental observations and is consistent with our recent graphical model (Romani et al., 2013).

## 1. Introduction

Human long-term memory capacity for names, facts, episodes and other aspects of our lives is practically unlimited. Yet recalling this information is often challenging, especially when no precise cues are available. A striking example of this deficiency is provided by classical studies of free recall, where participants are asked to recall lists of unrelated words after a quick exposure (Murdock, 1962; Kahana, 1996). Even for short lists of 5–10 words most of participants are unable to reproduce them without omissions (Murdock, 1960; Tulving, 1966).

Several influential models of recall were developed. Some of them were driven by the description of behavioral aspects (Glenberg and Swanson, 1986; Howard and Kahana, 1999; Davelaar et al., 2005; Brown et al., 2007); while others were rooted in biological principles (Grossberg and Stone, 1986; Ruppin and Yeshurun, 1991; Wong et al., 1991; Hasselmo and Wyble, 1997; Verduzco-Flores et al., 2012; Lansner et al., 2013).

According to the influential “search of associative memory” (SAM) model, items presented for recall acquire a set of mutual associations when stored temporarily in working memory buffers (Raaijmakers and Shiffrin, 1980). These acquired associations are then used to retrieve words from memory. SAM can be fit to reproduce recall data with great precision (Raaijmakers and Shiffrin, 1981), but since it has many parameters it cannot provide the first-principle explanation for very limited recall capacity observed in experiments. A recent model of memory retrieval (Romani et al., 2013; Katkov et al., 2015) introduced the notion that long-term associations between items determined by overlaps between their neuronal representations in memory networks, rather than short-term associations acquired during the experiment, are primarily responsible for recall process. With a simple phenomenological implementation of recall, this assumption results in a generic limit for the recall capacity compatible with the data (Romani et al., 2013). Moreover, the neuronal representations determine the recall probability of different items (“easy” vs. “difficult” words) and the order of their recall. In the current contribution, we develop a more realistic neural network model where recall is mediated by the sequential reactivation of neuronal ensembles encoding different items in memory. We show existence of stable states of the network corresponding to the activation of neuronal ensembles encoding single memory state and to the activation of intersection of neuronal ensembles encoding two or more memory states. We identify these different phases of the model with mean-field analysis of the network dynamics. We, further, show that the transitions between the memories may be driven by periodic modulation of the feedback inhibition that pushes the network to oscillate between the attractor memory states and intersections between these states, as was suggested in Romani et al. (2013). We identify these different phases of the model with mean-field analysis of the network dynamics. Based on this analysis we perform extensive numerical simulations to characterize the recall behavior of the model. In addition, we modeled short-term associations between memory items formed during the acquisition and characterize their effects. Finally, we systematically characterize the effects of neuronal noise on recall. The main goal of this paper is to present the network model of recall compatible with well-known features of free recall observed over many years of research. Some of the predictions of the model are also tested against a large recent dataset of free recall that was collected and made available by the lab of Prof. Kahana from University of Pennsylvania (see details in Section 2).

## 2. Materials and methods

### 2.1. The dynamics

We consider a Hopfield neural network of *N* rate-neurons (Hopfield, 1984, see also Grossberg, 1988). The dynamics of neuron *i* is represented by the equation:

(1) $$\tau {\dot{c}}_{i}(t)=-c_{i}(t)+\sum_{j=1}^{N}J_{ij}\;\cdot \;r_{j}(t)+\xi_{i}(t),$$

(2) $$r_{i}=g(c_{i})\;.$$

where *c*, *r* are respectively the synaptic currents and the firing rates, *J* the connectivity matrix, each **ξ**_*i*_ is an independent random variable having a gaussian distribution with mean zero and variance ξ_0_ and τ is a constant^1^.

The gain function is:

(3) $$\{\begin{array}{l} g={\left(x+\theta \right)}^{\gamma }\;x+\theta >0\;,\\ g=0\;x+\theta \leq 0\;. \end{array}$$

where θ > 0 is a threshold for the activation of a neuron while γ defines the gain and is constrained to γ < 1 for the gain function to be sublinear.

Each of the *P* memory items is represented by bynary vectors of *N* bits:

(4) $$\eta^{\mu \in \{1..P\}}=\underset{\text{N neurons}}{\underset{︸}{100011101001..1001}}\;.$$

where each bit has an indipendent random binary value, being 1 with probability *f* and 0 with probability 1 − *f* (Kanerva, 1988; Treves and Rolls, 1991). We use these vectors to define the connectivity matrix *J* according to the Hebbian rule (Tsodyks, 1989):

(5) $$J_{ij}=\frac{\kappa }{N}\left(\sum_{\mu =1}^{P}\left(\eta_{i}^{\mu }-f)(\eta_{j}^{\mu }-f\right)-\varphi \right)\;.$$

where κ and φ are two parameters that respectively define the strength of excitation and the relative strength of inhibition in the network. When simulating the network, all parameters are held constant except for the relative strength of the inhibition φ.We say that a particular memory is “recalled” when the corresponding memory pattern is active.

Memory μ is recalled if the average firing rate of neurons corresponding to memory μ (*i* such that $\eta_{i}^{\mu }=1$) is above the threshold value *r*_*thresh*_. This threshold is chosen so that two memories are never recalled simultaneously. If in a given time interval, e.g., from time 0 to *T*, the state of the network was in memories μ_1_, μ_2_, μ_3_.. at different times, we say that the network has “retrieved” these memories in a time *T*.

A slight modification of the model allows to account for short-term associations as in the SAM model. For example, temporal contiguity is the tendency to recall neighboring presented items in temporal proximity. To account for this effect we add a new term to the connectivity matrix *J*_*ij*_:

(6) $$J_{ij}^{+-}=J_{ij}+\delta J_{ij}=J_{ij}+J_{+}\sum_{\mu =1}^{P-1}\eta_{i}^{\mu }\eta_{j}^{\mu +1}+J_{-}\sum_{\mu =2}^{P}\eta_{i}^{\mu }\eta_{j}^{\mu -1}.$$

The new part δ*J*_*ij*_ consists of two terms which respectively connect a given memory μ with the memories presented immediately before and after it (μ − 1 and μ + 1) (Sompolinsky and Kanter, 1986; Griniasty et al., 1993). In doing so the memories are chained one to the other in the ‘forward’ and ‘backward’ direction with an asymmetry which depends on the values of *J*_+_ and *J*_−_.

### 2.2. Meanfield theory

We analyze the network in the absence of noise (ξ_0_ = 0) and temporal contiguity (*J*_+_ = *J*_−_ = 0). To quantify the degree of memory activations we introduce the “overlaps” defined as in Amit and Tsodyks (1991):

(7) $$\{\begin{array}{l} m^{\mu }(t)=\frac{1}{N}\sum_{i=1}^{N}\left(\eta_{i}^{\mu }-f)r_{i}(t\right)\;,\;\mu \in \{1..P\}\\ m^{0}(t)=\frac{1}{N}\sum_{i=1}^{N}r_{i}(t)\;. \end{array}$$

While *m*^0^(*t*) measures the average firing rate in the network at time *t*, each *m*^μ^(*t*) measures the difference between the average firing rate of neurons encoding memory μ and all other neurons:

(8) $$\begin{array}{l} m^{\mu }(t)=\frac{1}{N}\sum_{i=1}^{N}\left(\eta_{i}^{\mu }-f)r_{i}(t\right)=\\ \;=\frac{1}{N}\sum_{i=1}^{N}\left(\left(1-f)\eta_{i}^{\mu }r_{i}(t)-f(1-\eta_{i}^{\mu })r_{i}(t\right)\right)=\\ \;=(1-f)f\sum_{i=1}^{N}\frac{\eta_{i}^{\mu }r_{i}(t)}{fN}-\frac{\left(1-\eta_{i}^{\mu })r_{i}(t\right)}{(1-f)N}\;. \end{array}$$

At a fix point of the network dynamics (Equation 2) the synaptic currents can be expressed via the values of the overlaps:

(9) $$c_{i}=\sum_{j=1}^{N}J_{ij}r_{j}=\sum_{\mu =1}^{P}\kappa ((\eta_{i}^{\mu }-f)m^{\mu }-\varphi m^{0})\;,$$

(10) $$r_{i}=g(c_{i})\;;$$

given by Equation (2) in Equation (7). This shows that one can calculate *r* for each neuron *i* given the set of *m*′*s*. Pluggin Equation (10) into Equation (7) we obtain a a system of *P* + 1 equations for the overlaps *m*′*s*. The solutions to such a system are the possible fixed points of the network. Consider a vector $\eta_{i}\in {\left\{0,1\right\}}^{P}$ representing the encoding of each memory item by neuron *i*. There are 2^*P*^ possible realizations of vector **η**_*i*_ that are denoted by a random vector *v* ∈ {0, 1}^*P*^ where each component is indipendent from any other being 1 with probability *f* and zero otherwise. Each realization of *v* identifies a population of neurons. We say that neuron *i* belongs to a population *v* if **η**_*i*_ = *v* that is $\eta_{i}^{\mu }=v^{\mu }\;\forall \mu$. Furthermore, we say that a population *v* belongs to a memory μ if *v*^μ^ = 1 (Curti et al., 2004).

The cardinality of a vector is defined as

(11) $$|v|=\sum_{\mu }v^{\mu }\;.$$

The probability for each vector *v* is:

(12) $$S_{v}={\left(1-f\right)}^{P-|v|}\cdot f^{\left|v\right|}\;,$$

while the synaptic current for each neuron in population *v* is:

(13) $$c_{v}=\sum_{\nu =1}^{P}\kappa ((v^{\nu }-f)m^{\nu }-\varphi m^{0})\;.$$

The fixed point solutions can then be characterized in the limit *N* → ∞ in terms of these population vectors. Plugging Equation (10) into Equation (7) and summing up we obtain in the limit *N* → ∞:

(14) $$\{\begin{array}{ll} m^{\mu }= & {\langle (v^{\mu }-f)\cdot g\left(c_{v}\right)\rangle }_{v}\\ m^{0}= & {\langle g\left(c_{v}\right)\rangle }_{v}\;. \end{array}$$

where the average can be expressed in terms of the probability *S*_*v*_ as:

(15) $$\{\begin{array}{ll} m^{\mu }= & \sum_{v}\left(v^{\mu }-f\right)S_{v}\;\cdot \;g\left(c_{v}\right)\\ m^{0}= & \sum_{v}S_{v}\;\cdot \;g\left(c_{v}\right) \end{array}$$

This system determines the fixed points of the network in the meanfield limit. It cannot be solved in general but for a given *ansatz* of the solution it is possible to determine the region, in the parameter space, for its existence and stability. The type of solutions that we analyze are those that represent either a single memory or the intersection between memories. The correct *ansatz* for these solutions are easily expressed in terms of the synaptic currents. A single memory solution is then defined by the following conditions:

- the currents to each population *v* that belongs to the active memory μ are uniformly above threshold *c*_*v*_ + θ > 0 if *v*^μ^ = 1;
- the currents to each population that doesn't belong to the active memory μ are below threshold *c*_*v*_ + θ < 0 if *v*^μ^ = 0;

This two conditions define our ansatz for a single memory state. From this definition it follows that in the state of single memory the the only overlap *m* different from zero is the one of the active memory *m*^μ^. Similarly we define the ansatz for the intersection between two or more memories. In this state only two overlaps *m* are different from zero. For each of these ansatz one can find its region of existence and stability in parameter space. In such a region the solution is steady state of the system. A detailed theoretical analysis of these regions goes beyond the scope of this paper and will be presented in a future publications.

### 2.3. Simulation technique

To study the influence of finite size effects and noise on the dynamics of the network we simulate the dynamic of a network of *N* = 10^5^ neurons. To achieve this goal we simplify the system in Equation (2). This is a dimensionality reduction of the network that reduces the number of simulated units. All the neurons that have the same vector **η**_*i*_ (i.e., are in the same population *v* such that **η**_*i*_ = *v*) can be described by a single unit. For these neurons the afferent connections given by the matrix J are identical. Each neuron receives the same input and projects equally on other neurons. It is not possible to differentiate their activity except for the effect of the noise term **ξ**. But in Equation (2) we can average terms which share the same connections averaging also their noise. For a given realization of the network we can write the fraction of neurons in a given population *v* as:

(16) $$S_{v}=\frac{1}{N}\times \{number\;of\;i\;such\;that\;\eta_{i}=v\}\;,$$

which converges to the definition of Equation (12) in the limit of *N* → ∞. Defining *c*_*v*_(*t*), the averaging synaptic current *c*(*t*) for a neuron in population *v* at time *t*, it is then possible to write an equation for the dynamics of *c*_*v*_(*t*). By summing Equation (2) over all neurons which belong to the same population *v* we obtain:

(17) $${\dot{c}}_{v}(t)=-c_{v}(t)+\sum_{w}{\tilde{J}}_{vw}\;\cdot \;S_{w}\;\cdot \;g(c_{w}(t))+{\tilde{\xi }}_{v}(t)\;,$$

where ${\tilde{\xi }}_{v}$ is a gaussian white noise with mean zero and amplitude ${\tilde{\xi }}_{v}=\xi_{0}\cdot S_{v}\cdot N$, while ${\tilde{J}}_{vw}$ is given by:

(18) $$\begin{array}{l} {\tilde{J}}_{vw}=\frac{\kappa }{N}\sum_{\mu =1}^{P}((v^{\mu }-f)(w^{\mu }-f)-\varphi )+J_{+}\sum_{\mu =1}^{P-1}v^{\mu }w^{\mu +1}\\ \;+J_{-}\sum_{\mu =2}^{P}v^{\mu }w^{\mu -1}. \end{array}$$

The vectors *v* and *w* are binary vectors of length *P* identifying different populations. The system of Equation (17) is a reduction of the original system of Equation (2), it has 2^*P*^ equations instead of the *N*. In this reduction the only piece of information which is not accessible is the precise value of the firing rate of each single neuron. Only the average firing rate of the population it belongs to is now accessible. The actual number of equations to simulate depends on the particular realization of the network given by the choice of η^μ∈{1..*P*}^. Although in principle the system has 2^*P*^ equations, in practice, due to the finite size of the network and its sparse connectivity, there are much less populations since *S*_*v*_ = 0 for most *v* (Curti et al., 2004). The total number of equations in the system will depend on *N* and *f* but will always be less than *N*, tending to *N* only for very large *P*. In this framework, for *P* = 16, we are able to simulate easily a large network of *N* = 10^5^ neurons. Indeed taking *f* = 0.1, the number of equations to simulate drops from the 10^5^ of the original system in Equation (2) to the ≈1000 of the reduced one of Equation (17).

Simulations are run according to Equation (17) employing the parameters in Table 1. The number of simulated networks is *N*_*trials*_. For each simulation the network is initialized in the state of a single, randomly chosen memory μ. In this state all the populations *v* which belong to memory μ are initialized to a rate *r*_*ini*_ while the others are initialized to a zero rate. In the model the transitions between memories are triggered by oscillations of the variable φ. This oscillates sinusoidally between the values φ^*max*^ and φ^*min*^. The oscillations have a period τ_*o*_ which is much larger than τ so that the network is undergoing an adiabatic process. Integrations of Equation (17) are performed with the Euler method with a time step of *dt* and the simulated interval is [0..*T*]. The total number of cycles of oscillations is *T*∕τ_*o*_.

**Table 1:** **Reference values for the parameters in the simulation**.

**Parameters and hyperparameters**		
**Name**	**Description**	**Value**
N	Number of neurons	100,000
P	Number of memories	16
f	Sparsity	0.1
τ	Decay time	0.01
κ	Excitation parameter	13, 000
φ^*max*^	Max inhibition parameter	1.06
φ^*min*^	Min inhibition parameter	0.7
γ	Gain function exponent	2/5
θ	Gain function threshold	0
τ_*o*_	Oscillation time	1
*T*_*tot*_	Total time	450
*dt*	Integration time step	0.001
*J*_+_	Forward contiguity	1500
*J*_−_	Backward contiguity	400
ξ_0_	Noise variance	65
*r*_*thresh*_	Recall threshold	15
*N*_*trials*_	Number of trials	10, 000
*r*_*ini*_	Initial rate	1

### 2.4. Experimental methods and data analysis

The data analyzed in this manuscript were collected in the lab of M. Kahana as part the Penn Electrophysiology of Encoding and Retrieval Study. Here we analyzed the results from the 141 participants (age 17–30) who completed the first phase of the experiment, consisting of 7 experimental sessions. Participants were consented according the University of Pennsylvanias IRB protocol and were compensated for their participation. Each session consisted of 16 lists of 16 words presented one at a time on a computer screen and lasted approximately 1.5 h. Each study list was followed by an immediate free recall test. Words were drawn from a pool of 1638 words. For each list, there was a 1500 ms delay before the first word appeared on the screen. Each item was on the screen for 3000 ms, followed by jittered 800–1200 ms inter-stimulus interval (uniform distribution). After the last item in the list, there was a 1200–1400 ms jittered delay, after which the participant was given 75 s to attempt to recall any of the just-presented items. Only trials without errors (no intrusions and no repeated recalls of the same words) were used in the analysis.

We analyze this dataset to validate our model. We investigated several aspects of the dataset as described in Katkov et al. (2014, 2015). Here we show the plots concerning semantic similarity in **Figures 5B,D**. Of all the trials we exclude those where items not belonging to the presented list were reported (intrusions) and those where at least one word was retrieved twice (repetitions). For each list we then associate to each pair of words their LSA score as obtained from online datasets. We then consider the pairs formed by orderly associating two consecutively reported items. For each of these pairs we obtain the transition rank by ranking the LSA score the pair among all the scores of the first item with any other word in the list. As there are 16 words the maximum rank is 15 and the minimum is 1. This is the quantity shown on the x-axis of **Figure 5B**.

For each pair of consecutive reported items we compute the IRT by the difference of their times of retrieval. This is the quantity shown on the y-axis of **Figure 5B** vs. the LSA score of the same pair.

## 3. Results

### 3.1. Meanfield theory vs. network simulations

**Figure 1:**
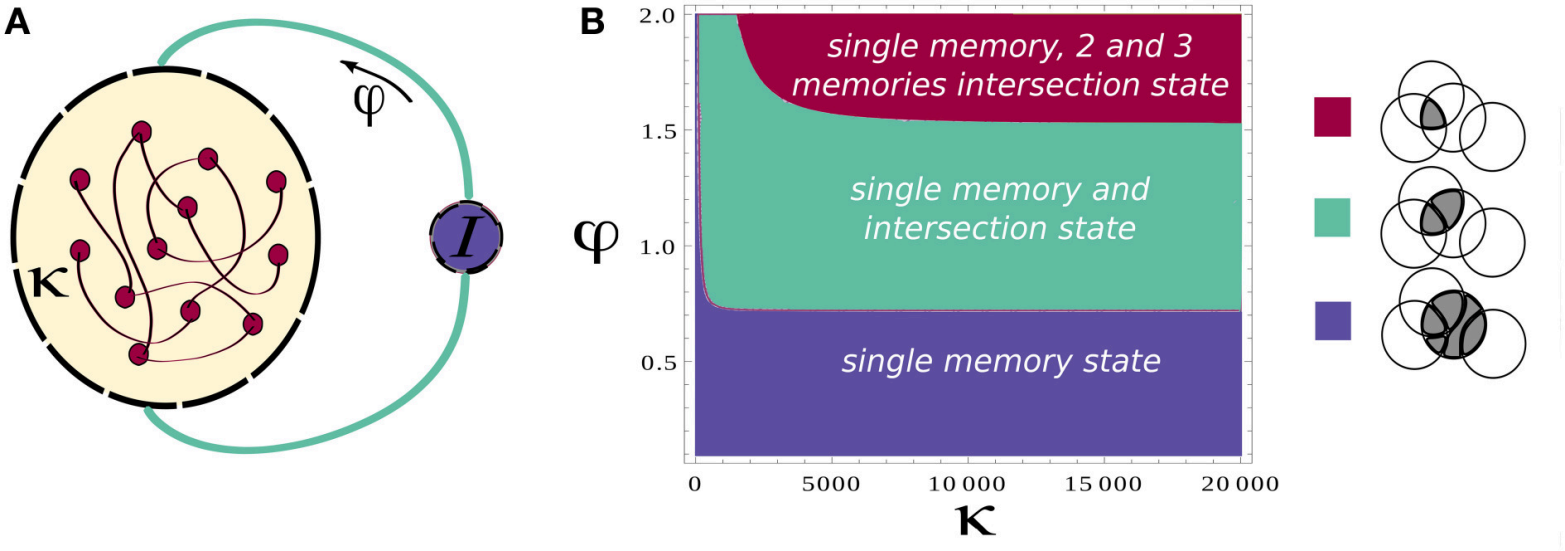
**Network architecture and Mean-field phase diagram. (A)** Neurons in the network are connected through simmetric connections induced by hebbian learning. Homeostatic control is induced by the inhibition strength determined by φ. **(B)** Mean-field phase diagram for the parameters κ and φ. The legend illustrates different phases. Circles denote a pool of neurons encoding a particular memory. For low values of φ the single attractor solution is found, as φ is increased other solutions appear. Parameters values are according to Table 1.

The main principle of recall that was suggested in Romani et al. (2013) is that externally generated control signal, expressed in periodic modulation of the strength of feedback inhibition, drives the network to oscillate between two states; one state is characterized by activation of single attractors, which correspond to a recall of the corresponding item (Hasselmo and Wyble, 1997; Gelbard-Sagiv et al., 2008; Romani et al., 2013); the second state is the intersection between pairs of attractors, which is a step toward transitions between different items. In this way each retrieved item acts as an internal cue for the next one (Raaijmakers and Shiffrin, 1981). Here we use the meanfield analysis of the network (see Section 2) to confirm that these two state types are indeed present. We identify the parameter regimes for their existence and stability. The meanfield theory greatly simplifies the analysis of the network by reducing the dynamics from that of single neurons (Equation 2) to overlaps, which are variables that describe the degree to which the network state corresponds to one of the memory attractors (see Equations 7 and 15 in Section 2). In the state of single attractors, only one overlap is positive while other ones are zeros. In the intersection states, pairs of overlaps are positive. We therefore use the meanfield equations that determine the possible values of overlaps (Equation 15) to find solutions corresponding to the intersection of *Q* memories. These solutions are characterized by *Q* positive overlaps: *m*^1^ = … = *m*^*Q*^ = *m*^*active*^. The overlaps have all the same values as all the active neurons in the intersection of *Q* memories fire at the same firing rate. The precise solution depends on the choice of the gain function in Equation (3). For concreteness, we chose a saturating gain function with threshold, with the exponent of γ = 1∕2 that allows analytical solution. The solution to Equation (15) is

(19) $$\{\begin{array}{ll} & m^{0}=\frac{1}{2}(k^{2}f^{2Q}\left({\left(f-1\right)}^{2}Q-\varphi \right)+\\ & \;\sqrt{k^{4}f^{4Q}{\left(\varphi -{\left(f-1\right)}^{2}Q\right)}^{2}+4\theta k^{2}f^{2Q}})\;,\\ & m^{active}=(1-f)\;\cdot \;m^{0}\;,\\ & m^{inactive}=0 \end{array}$$

where *m*^*active*^ and *m*^*inactive*^ are respectively the value of the overlap for an active and inactive memory and *m*^0^ denotes the average activity of the network. *f* denotes the sparseness of memory representations, *k* scales the strength of the recurrent associative synapses and φ defines the relative strength of inhibition, Figure 1A (see Section 2 for more details). The existence of these solutions requires the term in the square root to be positive, which results in the phase diagram shown in Figure 1B. Increasing the relative strength of feedback inhibition, the network state goes from the regime with only single attractor states to the one where single attractor and intersection of pairs of attractors coexist. More elaborated analysis of stability, which will be presented elsewhere, shows that these solutions are stable in the whole region of their existence, but the relative stability of single attractor states relative to the intersection states is decreasing with the increase in φ.

**Figure 2:**
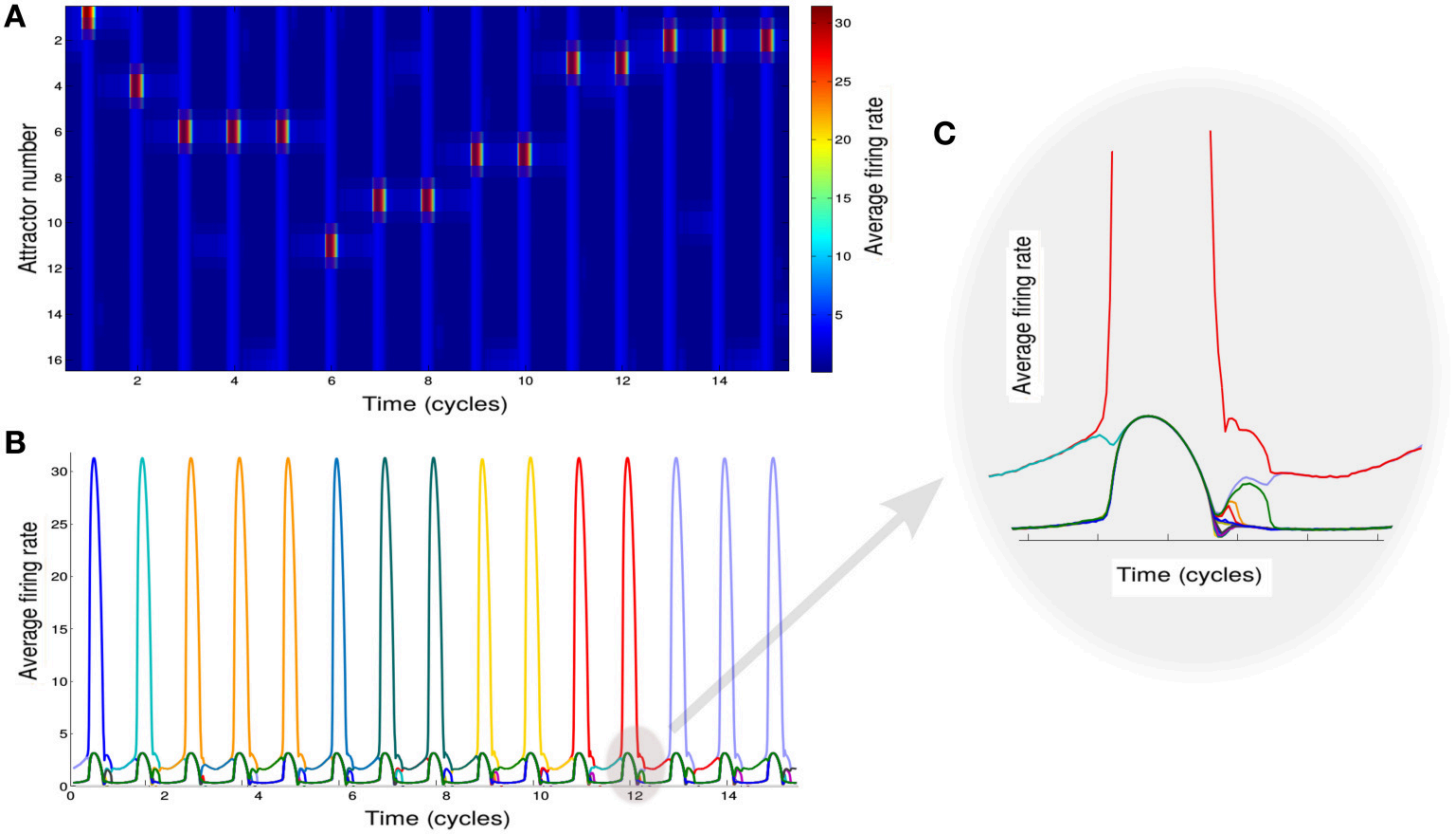
**Neural network activity. (A)** Activity of the attractors in the network. Different rows correspond to the average firing rate of different memories for 15 cycles of oscillation of φ. **(B)** Activity of the attractors in the network. Each colored line correspond to the average firing rate of a different memory. **(C)** Details of the neuronal dynamics.

Based on this analysis, we simulate the network while modulating the inhibition to cause the transitions between these two states (see Section 2 for details of simulations). We also add noise in order to trigger the transitions to the intersections between two attractors when inhibition rises. To mimic the experimental protocol (see Section 2), we simulate multiple recall trials where random samples of 16 items are selected for each trial. One sample epoch of simulations is shown in Figures 2A,B.

Each of the colored line in Figure 2B shows the average firing rate of neurons representing a specific memory. When one of these is above the threshold value of *r*_*thresh*_ we regard the corresponding memory as retrieved. We note that the precise sequence of retrieved items is not predictable for a given list of presented words, as it strongly depends on the first item being recalled (here assumed to be chosen randomly) and is sensitive to noise.

The effect of the oscillations is to modulate the overall activity in such a way that at each cycle the state of the network can potentially move from one attractor to another. The details of the underlying dynamics are shown in the plot of Figure 2C which zooms on the shadowed region in Figure 2B to show the transition from a single attractor to an intersection. This will lead to the retrieval of a new memory.

Although a switch between different states of the network is induced at every oscillation cycle, not always the state of the network shifts toward a new memory (Figures 2A,B). Rather it can remain in the same state or shifts toward an already explored memory so that only stochastically new memories are retrieved.

### 3.2. Time course of retrieval

**Figure 3:**
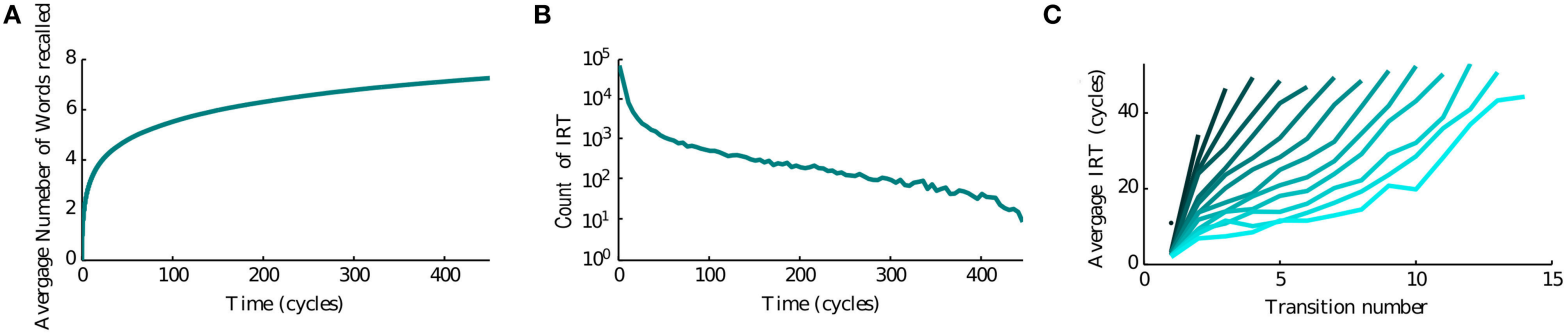
**Temporal properties of recall**. **(A)** Average number of words recalled vs. time. **(B)** Distribution of the IRTs. **(C)** IRT average (y axis) for ordered transitions between words (x-axis). Each line represents the average over the set of trials in which a different number of words were recalled: going from left to right (or dark to light blue) less to more words up to the maximum of 16.

Since the recall of subsequent memories is a stochastic process triggered by noise in the input, we perform multiple simulations to characterize the average accumulation of recalled memories with time (Figure 3A). We observe that after a quick initial accumulation, the retrieval process slows down sharply, however the number of memories recalled continues to increase. This behavior is compatible with experimental observations (Rohrer and Wixted, 1994; Wixted and Rohrer, 1994) and with results obtained by stochastic implementation of the free recall model presented in Katkov et al. (2015). The time between the recall of subsequent items (inter-retrieval time, IRT) is highly variable as shown in Figure 3B. Even after very long time-intervals it is possible to retrieve new items, in line with the experimental findings. We note that while the average accumulation curve is monotonic and smooth, each trial is characterized by a highly irregular set of IRTs, with short IRT interspersed between long ones due to cyclic transitions between items with relatively large overlaps. This is broadly consistent with experimental data (results not shown). Following the experimental study of Murdock and Okada (1970), Rohrer and Wixted (1994), we analyzed the average time progression of recall for trials with a certain number of words recalled (in a time window of 500 oscillation cycles). An interesting observation is that the corresponding curves separate already at the beginning of the recall, i.e., in the trials where more items are recalled eventually, the recall begins faster than in less successful trials, Figure 3C. This observation is also in line with the experimental results and with the stochastic model of Katkov et al. (2015).

### 3.3. Effects of long-term memory representations

**Figure 4:**
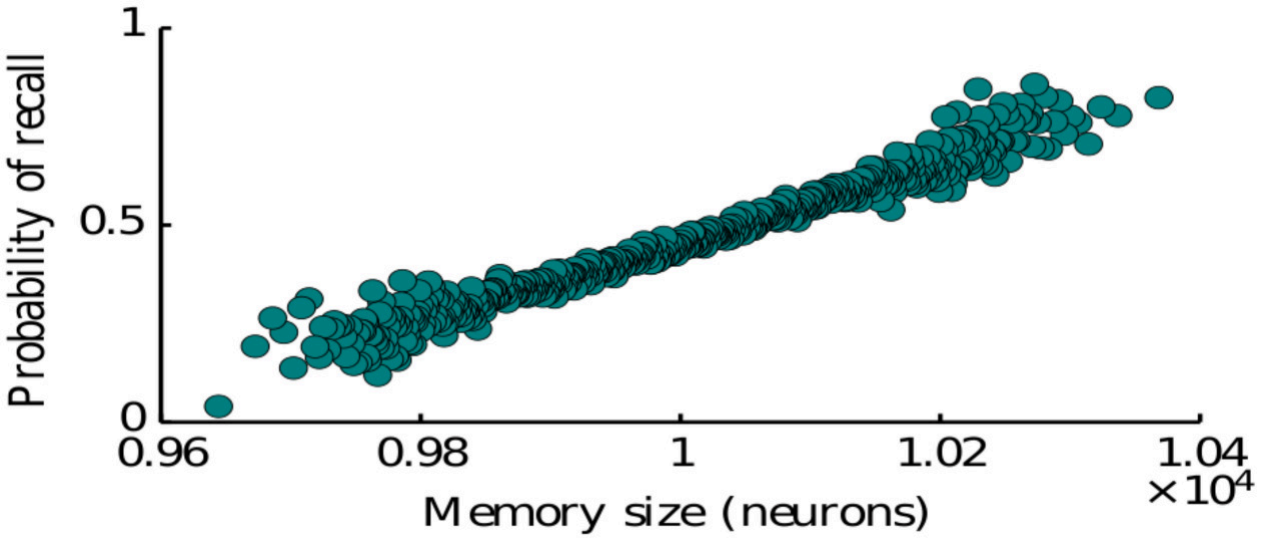
**Probability of recalling an item of a given size**. The size is the number of neurons encoding for that particular memory.

Here we study the dependence of the recall process on the statistics of memory representations as defined by the memory patterns introduced in Section 2 (see Equation 4). In particular we consider the effects of representation size (number of neurons encoding a given item) and the size of intersections between the representations of two memories (number of neurons encoding both of the items). The representation size higly influences the probability of recall for a given memory. Our simulations show that simulating the network many times with items having a randomly drawn size, the probability to recall an item is monotonically increasing with the size of the corresponding representation (Figure 4). This is predominantly due to the fact that items represented by more neurons have on average a larger intersections with other items, since we assumed random encoding. Indeed as we show below, the intersection sizes play a major role in determining the subsequent items to be recalled. Therefore, our model is in agreement with the graph model of Romani et al. (2013), Katkov et al. (2015) where items with larger representations have higher probability to be recalled (easy vs. difficult items).

**Figure 5:**
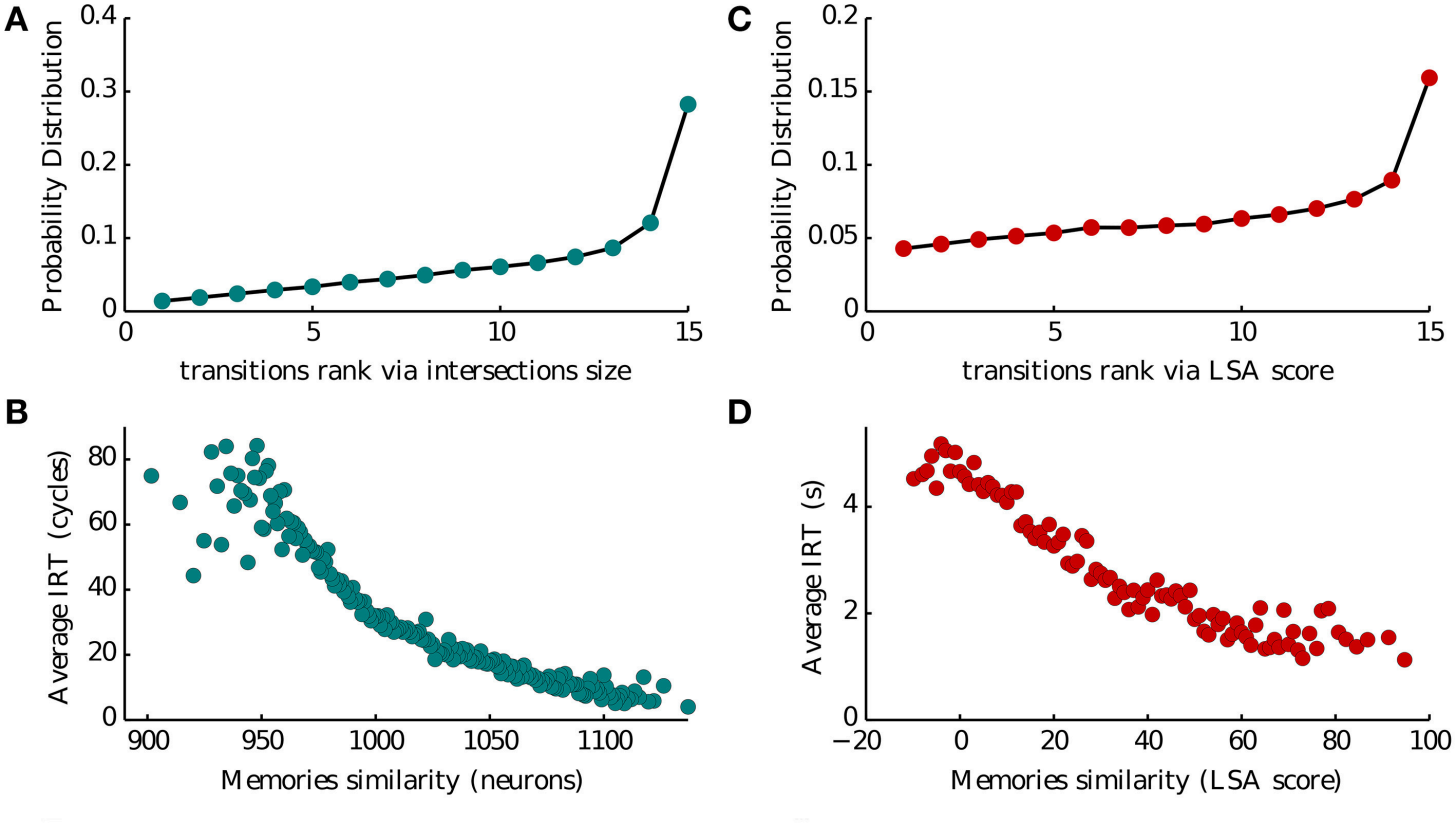
**Memory transitions. (A,C)** Probability density of transitions between two subsequent recalled memories as a function of the ranked size of their intersection (1–15 going from the less to the most similar) and of their Latent Semantic Analysis score (LSA). **(B,D)** Average IRT between two subsequent recalled memories as a function of the size of their intersection (in number of neurons) and of their Latent Semantic Analysis score (LSA).

Intersections between memory representations play a crucial role in our model of recall. In Romani et al. (2013) intersection sizes (the number of neurons encoding a pair of items) were assumed to govern the transitions between the recall of successive items. To evaluate the role of intersection sizes in the transitions between items we ranked intersection sizes for each presented list of 16 words, from low to high (1–15), and computed the probability of transition for each intersection rank (Figure 5A). Thirty percent of transitions occurred for largest intersection with the currently recalled item, the probability of other transitions monotonically decreases with the rank of intersections. Moreover, we found that the inter-recall time between the successive items also exhibited monotonic relation to the intersection size, with larger intersections leading to faster transitions (Figure 5C). These results indicate that the sizes of inter-item neuronal intersections to a large extent determine the temporal evolution of recall. It is therefore tempting to speculate that they are neuronal correlates of semantic similarity between the items (Baddeley, 1966; Mandler et al., 1969; Howard and Kahana, 2002b). To further elaborate on this hypothesis, we analyzed the dataset of free recall of lists of unrelated words collected and made available by Prof. Kahana from the University of Pennsylvania. We considered a measure of semantic similarity called (*Latent Semantic Analysis*, or LSA), which represent the number of times two words appear together in a representative corpora of natural text (Landauer and Dumais, 1997). We then used this measure to evaluate the effect of semantic similarity on the probability and speed of inter-item transitions in experimental observations, and obtained a remarkable agreement with the corresponding model predictions (compare Figures 5A,C with Figures 5B,D).

### 3.4. Performance

We now focus on factors which influence the recall performance, namely the number of items that can be retrieved in a given time window, between time 0 and time *T*. This window is chosen to be long enough such that the recall slowed down considerably (see Figure 3A). In particular we will consider the effects of temporal contiguity and noise.

**Figure 6:**
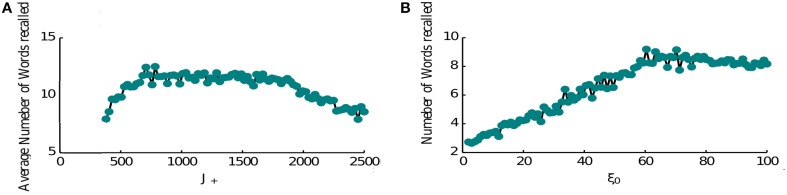
**Recall performance. (A)** Temporal contiguity and performance: average number of words recalled as a function of *J*_+_. *J*_+_ ranges between the fixed value of *J*_−_ = 400 and 2500. The number of memories is *P* = 16. The number of memories is *P* = 16. **(B)** Noise and performance: the average number of words recalled is plotted at the vary of noise variance ξ. A small amount of noise helps the retrieval process triggering transitions from memory to memory. For high noise levels the retrieval mechanism is hindered.

The performance of the network is limited as item representations that control the retrieval dynamics are random and hence same items are recalled numerous times before the network can retrieve a new memory. It is known however that the order of recall is not completely random, e.g., words that have neighboring positions in the list have a tendency to be recalled in close proximity (Sederberg et al., 2010). This phenomenon is known as temporal contiguity and we model it by adding a special term in the connectivity pattern that links neighboring items to each other favoring the transitions between them (see Section 2, Equation 6), thereby overcoming the effects of randomness. Hence when the forward contiguity term is stronger, the network retrieves more items (Figure 6A). Although if it is too strong it becomes the only mechanism for triggering a transition and the average number of items retrieved will be half of the total number (8 items in Figure 6A). Indeed in this regime the network retrieves all items that come after the random initial one. Once it retrieves the last presented item it keeps retrieving it. The loop of connectivities via the second last item, which strongly projects on it, prevents the activation of any other memory.

Another crucial element of the model is the noise that causes the recall dynamics to escape the short loops and retrieve new items. We thus computed the network performance for increasing noise levels (Figure 6B). As expected, the performance is very poor for low noise amplitudes and increases for higher amplitudes. This growth is terminated at some optimal level of noise, after which the number of recalled items is slowly decreasing. The reason for this behavior is that at high noise levels, the network does not converge to inter-item intersections at high levels of inhibition, rather to noisy mixtures of different memories, which results in less robust transitions to other items when inhibition is reduced (results not shown). Based on these observations, we propose that noise amplitude could be regulated during the retrieval phase (e.g., with neuromodulators that control cortical synchrony) in order to facilitate the recall of items from long-term memory.

## 4. Discussion

We presented a neural network model of information retrieval from long-term memory that is based on stochastic attractor dynamics controlled by periodically modulated strength of feedback inhibition. The model provides a more realistic implementation of the mechanisms behind associative recall based on neuronal representations of memory items, as proposed in Romani et al. (2013); Katkov et al. (2015). The network behavior is broadly compatible with some of the long-standing observations on free recall, in particular the slow-down of recall speed, highly variable inter-recall times and strong effects of semantic similarity between words.

In classical models of recall, such as SAM (Raaijmakers and Shiffrin, 1980) or TCM (Howard and Kahana, 2002a; Polyn et al., 2009), performance is mainly influenced by the temporal associations acquired during stimulus presentation. These effects were also considered in a possible network implementation (Bradski et al., 1994). In contrast, our model is based on long-term memory representations. Simple modification of the model (see Equation 6) allows to account for the effect of temporal contiguity (Sederberg et al., 2010). Therefore, we show that effects due to long-term memory representations and to presentation order can be implemented in a single neural network. It is important to note that effects due to long-term representations are masked by temporal association effects, being visible only in large data sets having many trials over lists composed of randomly selected words from a large preselected pool of words. In such datasets the same word is roughly uniformly distributed across temporal positions and their neighborhood words. Consequently, temporal association effects on the level of individual words are averaged out, and effects due to long-term representations become clearly visible. There are two major effects that historically were not considered neither experimentally nor in models: (1) intrinsic difficulty of words to be recalled—existence of “easy” and “difficult” words for recall; (2) masking of “difficult” words by “easy” words—“easy” words are statistically recalled earlier in the trial and suppress the recall of “difficult” words (Katkov et al., 2015). This work is a first attempt to implement a neural network that is taking into account long-term representation of memorized items.

Our network model is based on the basic assumption that when a word is recalled, a corresponding neuronal ensemble that represents this word in long-term memory is temporarily activated. The issue that we dont explicitly address is how the words that are presented for recall are selected, or primed and why other word representations are not reactivated (excluding rare instances of erroneous recall of words from previous lists). In the spirit of Kahanas TCM model (Howard and Kahana, 2002a), such a priming could be mediated by the excitation arriving from a separate “context” network where representation of the experimental setting is active throughout the recall trial. We therefore ignored the neuronal representations of words that are not in the list and considered a network with effectively very low “loading” level (*P* ≪ *N*). More realistic implementation of the model with high loading levels should be considered in future.

Another simplifying unrealistic assumption of the model concerns the statistics of long-term representations that are taken as random uncorrelated binary vectors of fixed average sparsity. Real statistics of word representations is not clear but can be safely assumed to be much more complicated, possibly reflecting the rich semantic associations between words and the frequency of their usage. With our assumptions, overlaps between different representations exhibit Gaussian distribution with variance to mean ratio decaying in the limit of infinitely large networks. Considering the effects of overlap distribution in this limit requires an extended mean-field analysis that will be presented elsewhere.

Very often the same attractor is repeatedly activated before noise causes the transition to a new one, and it can still be activated again at a later time. Since participants are instructed to only recall each word once, we assume that they suppress the report of a word after it is already recalled. In some experiments, subjects are explicitly instructed to report a word as many times as it comes to mind during a recall. Comparing the model to the results of such experiments could be of interest for a future work.

We considered modulated inhibition as a driving force for transitions between network attractors. Other mechanisms could potentially play this role, e.g., neuronal adaptation or synaptic depression. We believe that oscillatory mechanism is more plausible as it allows the system to regulate the transitions by controlling the amplitude and frequency of oscillations. The oscillations of network activity could correspond to increased amplitude of theta rhythm observed in human subjects during recall (Kahana, 2006; Osipova et al., 2006) and other types of working memory experiments (Tesche and Karhu, 2000; Raghavachari et al., 2001; Jensen and Tesche, 2002). The way we implemented feedback inhibition is not fully biologically plausible. Feedback inhibition in the cortex is mediated by several major types of interneurons (Markram et al., 2004). In particular, one type of interneurons (VIP), was proposed as a gateway for regulating the local inhibition since it receives inputs from remote cortical and subcortical regions and preferentially targets other types of interneurons (Pi et al., 2013). More realistic neural network models of recall should include this kind of inhibition.

At the current level of realism, we propose to view our model as a platform for further development of realistic neural network models of information retrieval and other related types of cognitive tasks. Future modifications should include effects of positional order on recall, or positional chunking, i.e., the tendency to divide the presented lists on groups of contiguous words (Miller, 1956; Gobet et al., 2001), as well as primacy (tendency to recall earlier words with higher probability, see e.g., Grossberg and Pearson, 2008), or effects obtained in serial recall, such as e.g., encoding gradient or similar tasks (Averbeck et al., 2002, 2003; Farrell and Lewandowsky, 2004), where participants are forced to recall items in presented order, implying stricter tests on temporal associations.

## Author contributions

MT and SR designed the study; SR developed and simulated the model; MT, MK, and SR performed a mathematical analysis, SR and MK performed data analysis; all the authors wrote the paper.

### Conflict of interest statement

The authors declare that the research was conducted in the absence of any commercial or financial relationships that could be construed as a potential conflict of interest.

## Footnotes

^1^Here and in the following a bold character, e.g., *c*, identifies the entire vector
